# Resource Allocation in Wireless Powered IoT System: A Mean Field Stackelberg Game-Based Approach

**DOI:** 10.3390/s18103173

**Published:** 2018-09-20

**Authors:** Jingtao Su, Haitao Xu, Ning Xin, Guixing Cao, Xianwei Zhou

**Affiliations:** 1 Department of Communication Engineering, University of Science and Technology Beijing, Beijing 100083, China; 18518788115@163.com (J.S.); xwzhouli@sina.com (X.Z.); 2 Institute of Telecommunication Satellite, China Academy of Space Technology, Beijing 100081, China; xinning7@sina.com (N.X.); caogx@sina.com (G.C.)

**Keywords:** power control, wireless energy transfer, IoT system, mean field Stackelberg game

## Abstract

The IoT system has become a significant component of next generation networks, and drawn a lot of research interest in academia and industry. As the sensor nodes in the IoT system are always battery-limited devices, the power control problem is a serious problem in the IoT system which needs to be solved. In this paper, we research the resource allocation in the wireless powered IoT system, which includes one hybrid access point (HAP) and many wireless sensor nodes, to obtain the optimal power level for information transmission and energy transfer simultaneously. The relationship between the HAP and the sensor nodes are formulated as the Stackelberg game, and the dynamic variations of the energy for both the HAP and IoT devices are formulated through the dynamic game with mean field control. Then the power control in the wireless powered IoT system is formulated as a mean field Stackelberg game model. We aim to minimize the transmission cost for each sensor node based on optimally power resource allocation. Meanwhile, we attempt to minimize the energy transfer cost based on power control. As a result, the optimal solutions based on the mean field control of the sensor nodes and the HAP are achieved through dynamic programming theory and the law of large numbers, and ε-Nash equilibriums can be obtained. The energy variations for both the sensor nodes and HAP after the control of resource allocation based on the proposed approach are verified based on the simulation results.

## 1. Introduction

Internet of Things (IoT), as the main pattern to link between people and things, has been employed in the logistics for tail after, to build industry IoT environments, and for academia research [1,2]. Through IoT techniques [3], it is easy for people to access and control the date generated by the sensors, to structure the IoT system. In the IoT system, because the sensors are always battery limited devices [4], one of the main concern faced by the mass sensors is the energy consumption problem. Although the technology of NB-IoT is introduced by 3GPP to achieve low energy consumption [5], efficient energy utility in the IoT system still a key challenge that is under active research.

The development of the techniques for energy harvesting and wireless power transfer provides a paradigm to solve the energy efficiency and consumption problems in the IoT system [3,6]. Through the energy harvesting circuit, the sensors can harvest energy from different energy sources [7], such as sun light, wind, etc. Radio frequency (RF) based wireless power transfer in IoT system [8,9], which is more reliable and controllable, is also drawn a lot of research interests [10]. 

In this paper, we focus on the energy consumption problem in IoT system with RF based wireless power transfer, to achieve optimally resource allocation. We try to use the mean field Stackelberg game theory [11] to solve the resource allocation problem in the wireless powered IoT system, which consists of one HAP and a large number of sensor nodes. The mean field game is firstly inspired by [12,13], to solve the resource allocation problem with a large number of game players. In this paper, through combining the Stackelberg game and the mean field game, we aim at finding the optimal power control strategies when there is a large number of the sensor nodes. The dynamic characteristics of the battery’s energy variation is also considered in the proposed game model. We pay attention to the power control problem, to minimize the utility for both the HAP and the senor nodes. 

In summary, the key contributions of this paper are as follows:

- Firstly, we research a wireless powered IoT system, which consists of one hybrid access point (HAP) and N sensor nodes. The HAP is both the information collection center and the energy source for the sensor nodes.
- Secondly, a mean field Stackelberg game model is proposed to formulate the resource allocation problem in the proposed IoT system. The Stackelberg game is a one-leader-many-followers Stackelberg game. The HAP is the leader, where the sensor nodes are followers. For the mean field game, we use the energy variations as the system state. The objectives for the sensor nodes are to minimize the transmission cost during the energy transfer and information transmission. The objective for the HAP is to control the power level for energy transfer to minimize its utility.
- Finally, the mean field control for both the sensor nodes and the HAP are given based on dynamic programming and the law of large number. The ε-Nash equilibriums are also obtained and discussed.

The paper is organized as follows: Section 2 summarizes the related works. Section 3 gives the system model and problem formulation for the power control problem. Section 4 provides the mean field control of the sensor nodes with ε-Nash equilibrium, the mean field control of the HAP with a ε-Nash equilibrium. The implementation algorithm are also given in this section. Section 5 is the performance evaluation for both the sensor nodes and the HAP. Finally, the paper is concluded in Section 6.

## 2. Related Works

Although the battery limited problem can be solve through the wireless power transfer technique [14], the resource allocation problem in the wireless powered IoT system, especially the power control problem, is still an urgent problem that desperately needs to be solved. A large number of works have been done in this area [15,16,17,18]. In [15], the authors solve the resource allocation problem in cyber-physical IoT to maximize the energy efficiency. The proposed resource allocation scheme is based on the mixed integer non-convex programming theory and can be divided into two sub-problems, the power allocation problem and the channel allocation problem, and the Dinkelbach’s algorithm is used to solve the proposed optimal allocation problems. 

In [16], a green resource allocation method is proposed, which considers the QoE as the main influencing factor. Then the authors use the deep reinforcement learning to solve the QoE based resource allocation problem. 

In [17], a utility-lifetime maximization problem is considered for resource allocation. The authors use the Lagrange multiplier method to solve the proposed distributed dual subgradient algorithm. The wireless energy harvesting, wake-up radio and error control coding are all considered in model formulation. 

In [18], the authors formulate a distributed power control problem in the wireless powered communication networks as a utility maximization problem, to guarantee the QoS demand and to achieve efficient energy management. In this paper, the authors propose an energy-efficient communication approach considering both the WET and WIT phase. The optimal charging power for the energy source can be determined. 

The resource allocation problem in wireless powered IoT system has been considered and researched by lots of academies, but most of the previous works do not consider the size of the IoT system. When there are mass sensors in the IoT system, it is difficult to obtain the optimal power control strategy for each sensor. Meanwhile, the dynamic variation of the battery’s energy of the sensor node will also affect the resource allocation strategies, which is also not considered in the previous works.

## 3. System Model and Problem Formulation

### 3.1. System Model

**Figure 1:**
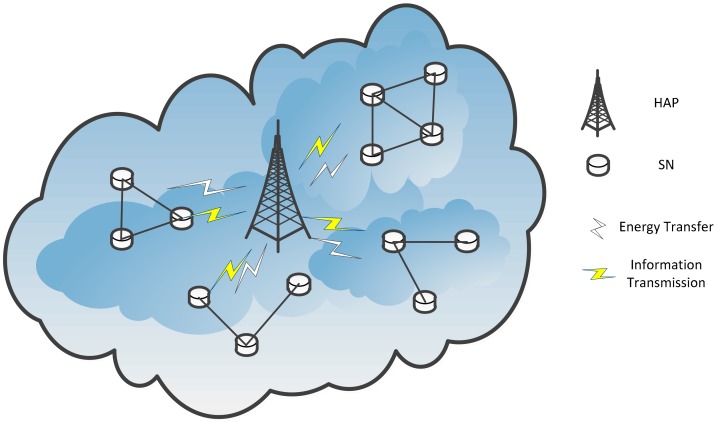
Wireless powered IoT system.

**Figure 2:**
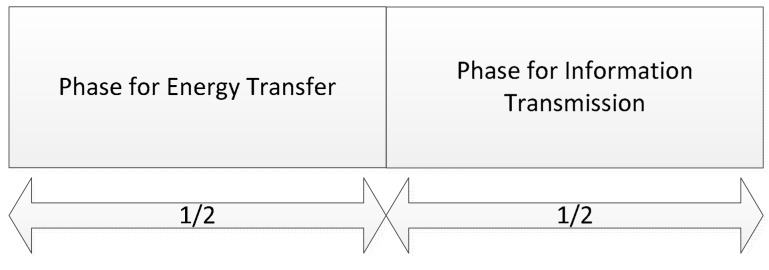
Time-switching protocol.

In this paper, we consider a wireless powered IoT system with one dedicated hybrid access point (HAP) and N sensor nodes (SNs). The system model is given in Figure 1. Located at the appropriate place, the HAP can be considered as an aggregation to collect information from the sensor nodes, and can be considered as an energy source to the sensor nodes through RF-based wireless energy transfer. Each SN should upload the information to the HAP, and harvest energy from the HAP using the equipped energy harvesting circuit. As each SN’s energy is limited by the battery capacity, it mainly uses the energy from the HAP for information transmission. Assuming that the energy transfer and information transmission can be done simultaneously. For the sensor nodes, we assume that the wireless energy and information transmission are operated at the same frequency, based on the “harvest-then-transmit” protocol [19,20], as shown in Figure 2. Based on the system model, we will try to find out the optimized allocated power levels for the resource allocation problems in the propose system. For the HAP, the optimal power strategy for energy transfer should be solved. For the SNs, the power solutions for information transmission are in demand. 

### 3.2. Stackelberg Game Framework

**Figure 3:**
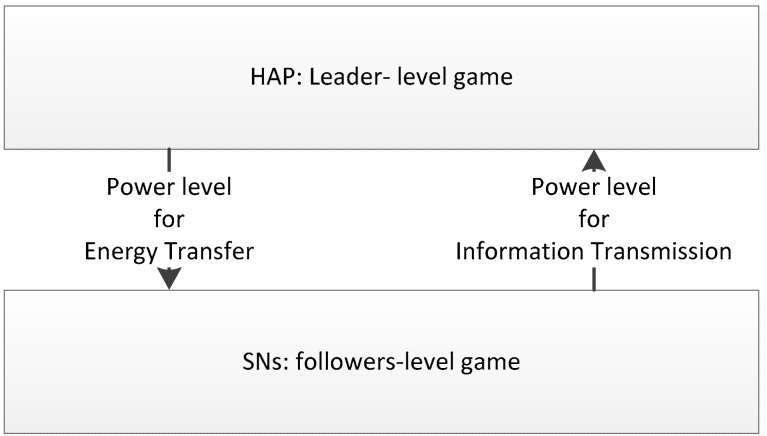
Stackelberg game.

In our proposed wireless powered IoT system, there are one dedicated HAP and N SNs. In the downlink scenario, the HAP controls its power level for energy transfer. In the uplink scenario, each SN controls its power level for information transmission. As each SN uses the energy from the HAP for information transmission, the power level for energy transfer can significantly affect the performance of the SNs. Then the relationships between the HAP and the SNs can be considered as a Stackelberg game, more specifically, it can be considered as a one-leader-many-followers Stackelberg game. The HAP works as the leader, where the SNs are the followers. The Stackelberg game is composed by two parts, the leader-level game and the followers-level game, respectively, as shown in Figure 3. 

(1) Leader-level game: As the HAP can significantly affect the performance of the SNs, it is considered as the leader. The HAP will transfer the energy to the SNs based on its own aspiration, and announces its strategy of power level for energy transfer to the SNs. Then the HAP can affect the SNs on their strategies for information transmission. Once the SNs make decisions on power level for information transmission, the HAP can re-adjust its energy transfer strategy to get more utilities.

(2) Follower-level game: As the SNs are affect by the HAP, they can be considered as the followers of the game. The SNs control their power for information transmission under the HAP’s energy transfer strategy, by playing a Stackelberg game. 

### 3.3. System State

In this paper, as we concentrate on controlling the power for both the HAP and SNs, we use the energy as the system state for mean field game (MFG) construction. There are two energy state variables for the proposed IoT systems, the energy level of the HAP denoted by $x_{0}\left(t\right)$, and the energy level of SN i denoted by $\left\{x_{i}\left(t\right),1\leq i\leq N\right\}$.

(1) Energy of the HAP: the energy of the HAP is mainly dominated by the power level for energy transfer. Assuming the energy is transferred by the HAP in a unique frequency, to avoid interference to information transmission and the power level for energy transfer is denoted by $p_{0}\left(t\right)$ at time instant t. For the HAP, the energy level can be described by the following differential equation:

(1) $$dx_{0}\left(t\right)=\left[\alpha_{0}x_{0}\left(t\right)+\beta_{0}p_{0}\left(t\right)\right]dt$$

where $x_{0}\left(t\right)$ is the energy level of the HAP, with an initial energy state $x_{0}\left(0\right)$. $\alpha_{0}$ is a random coefficient of energy degradation brought by the system consumption, an $\alpha_{0}x_{0}\left(t\right)$ denotes the energy brought by the system consumption. Generally, $\alpha_{0}x_{0}\left(t\right)$ can be represented as [21,22]:

$$\alpha_{0}x_{0}\left(t\right)=\left(P_{HC}+P_{RF}+P_{RP}\right)\delta_{0}$$

where $P_{HC}$ is the power consumption of the hardware circuit, $P_{RF}$ is the power consumption of the RF module, and $P_{RP}$ is the power consumption of packets exchanged by the HAP with controller. $\delta_{0}$ is the duration/slot for the energy transfer.

$\beta_{0}$ is a random efficiency coefficient of energy transfer, which depends on the energy transfer circuit. The energy transfer process should be a broadcast process. The initial state of the HAP is independent of the SNs with mean $Ex_{0}\left(0\right)={\bar{x}}_{0}$.

(2) Energy of the SNs: the energy of each SN is dominated by the energy from the HAP and the power for information transmission. Assuming the power for information transmission is denoted by $\left\{p_{i}\left(t\right),1\leq i\leq N\right\}$. For any specific SN, the evolution of the energy is described by:

(2) $$dx_{i}\left(t\right)=\left[\alpha_{i}x_{i}\left(t\right)+\beta_{i}p_{i}\left(t\right)+\rho_{i}h_{i}p_{0}\left(t\right)\right]dt$$

where $\left\{x_{i}\left(t\right),1\leq i\leq N\right\}$ is the energy level of the SN i. Each SN has an initial energy state, which is denoted by $\left\{x_{i}\left(0\right),1\leq i\leq N\right\}$, which are independent of each other with mean $\left\{Ex_{i}\left(0\right)=\bar{x},1\leq i\leq N\right\}$. In the current analysis, N is taken to be large so that MFG analysis may be applied. $\alpha_{i}$ is a random coefficient of energy degradation caused by the system consumption, which includes the power consumption of the hardware circuit and the RF module [23]. The power consumed in sensing and processing are also included in this coefficient [24]. $\beta_{i}$ is a random efficiency coefficient of information transmission, which depends on the information transmission circuit. $\rho_{i}$ is the conversion efficiency coefficient of energy transfer, and $h_{i}$ is the channel power gain from the HAP to SN i.

### 3.4. Problem Formulation

In this sub-section, we will give the optimal power control problem for the HAP and the SNs. We want to find the optimal power level for both the HAP and the SNs based on the proposed model. For the HAP, the optimal power strategy for energy transfer could be obtained by minimize the following utility function, which is:

(3) $$J_{0}^{N}\left(p_{0},p^{N}\right)=\int_{0}^{T}\left\{\mu_{0}{\left(x_{0}\left(t\right)-H_{0}x^{N}\left(t\right)\right)}^{2}+\nu_{0}{\left(p_{0}\left(t\right)\right)}^{2}\right\}dt$$

where $\mu_{0}\geq 0$ and $\nu_{0}>0$, are positive weighting factors representing relative importance of the objective components. The objective of the HAP is a linear combination of two components. The first component is the utility function denoted by $\mu_{0}{\left(x_{0}\left(t\right)-H_{0}x^{N}\left(t\right)\right)}^{2}$, which means the available energy for transfer, compared to the mass behavior of the energy of SNs. In the first component, $x^{N}\left(t\right)=\left(1/N\right)\sum_{i=1}^{N}x_{i}\left(t\right)$ denotes the mean field term that captures the mass behavior of the SNs. The second part is the payment earned from the SNs for energy transfer, and is denoted by $\nu_{0}{\left(p_{0}\left(t\right)\right)}^{2}$. Therefore, minimize the utility function of the HAP gives us the following objective function:

(4) $$p_{0}^{*}\left(t\right)=\underset{p_{0}\left(t\right)}{\arg\min}\;J_{0}^{N}\left(p_{0},p^{N}\right)$$

For the SNs, we want to find the optimal power strategies for information transmission considering a large population. Then, for any specific SN, its cost function is given as follow:

(5) $$J_{i}^{N}\left(p_{i},p_{-i},p_{0}\right)=\int_{0}^{T}\left\{\mu_{i}{\left(x_{i}\left(t\right)-H_{i}x^{N}\left(t\right)\right)}^{2}+\nu_{i}{\left(p_{i}\left(t\right)\right)}^{2}+\eta_{i}p_{0}\left(t\right)p_{i}\left(t\right)\right\}dt$$

where $\mu_{i}\geq 0$, $\nu_{i}>0$ and $\eta_{i}>0$ are positive weighting factors. The objective for any specific SN is composed by three parts. The first part is $\mu_{i}{\left(x_{i}\left(t\right)-H_{i}x^{N}\left(t\right)\right)}^{2}$, which means the available energy for information transmission, compared to the mass behavior of the SNs. The second part of the objective is $\nu_{i}{\left(p_{i}\left(t\right)\right)}^{2}$, denotes the power cost component for information transmission. The third part of the objective is $\eta_{i}p_{0}\left(t\right)p_{i}\left(t\right)$, which is the payment for the energy harvesting, depends on both the harvested energy and the power for information transmission. Therefore, minimize the objective for any specific SN gives us the following objective function:

(6) $$p_{i}^{*}\left(t\right)=\underset{p_{i}\left(t\right)}{\arg\min}\;J_{i}^{N}\left(p_{i},p_{-i},p_{0}\right)$$

For both the HAP and the SNs, the objective functions are formulated with the mean field game framework through the mean field term $x^{N}\left(t\right)$. Based on the mean field term, we can analyze the IoT system with a large population. Both the HAP and the SNs can obtain their distributed equilibriums by the estimation of the mass response.

## 4. Game Analysis and Implementation Algorithm

### 4.1. Mean Field Control of Sensor Nodes

In this section, we will try to get the mean field control solutions for the SNs, based on an energy transfer power strategy of the HAP. First, the local optimal control of each sensor node can be considered as a dynamic game and the open-loop and state feedback solutions will be given based on the Bellman’s dynamic programming principle. Then we will extend the size of the IoT system, and use the strong law of large numbers (SLLN) to get the mean field control solution for all the SNs, then each sensor node can obtain the distributed equilibrium solution based on the mean field control solution.

For each SN, it always constitutes an ε-Nash equilibrium for any control strategy of the HAP, which gives out the optimality of the optimal control problem for each SN, and is given as follows.

**Definition** **1** ( ε**-Nash equilibrium**).  *Given an energy transfer power strategy of the HAP, which is denoted by*$p_{0}$*, for each sensor node*{i,1≤i≤N}*, it constitutes an*ε*-Nash equilibrium, if there exists*ε≥0*such that*$J_{i}^{N}\left({\bar{p}}_{i},{\bar{p}}_{-i},p_{0}\right)\leq \inf_{p_{i}\in U_{i}\left(p_{0}\right)}J_{i}^{N}\left(p_{i},{\bar{p}}_{-i},p_{0}\right)+\varepsilon$*, for all*i*,*1≤i≤N.

**Proposition** **1.**  *For the sensor node*{i,1≤i≤N}*, a set of controls*$\left(x_{i}^{*},p_{i}^{*}\right)$*constitutes an open loop equilibrium to the power control problem in Equations (2) and (6), and the optimal control solution can be given by*:

(7) $$p_{i}^{*}\left(t\right)=-\nu_{i}^{-1}\beta_{i}\lambda_{i}\left(t\right)-\nu_{i}^{-1}\eta_{i}p_{0}\left(t\right)$$

*subject to*:

(8) $$\begin{array}{l} dx_{i}^{*}\left(t\right)=\left[\alpha_{i}x_{i}^{*}\left(t\right)+\beta_{i}\left(-\nu_{i}^{-1}\beta_{i}\lambda_{i}\left(t\right)-\nu_{i}^{-1}\eta_{i}p_{0}\left(t\right)\right)+\rho_{i}h_{i}p_{0}\left(t\right)\right]dt\\ =\left[\alpha_{i}x_{i}^{*}\left(t\right)-\nu_{i}^{-1}\beta_{i}^{2}\lambda_{i}\left(t\right)-\nu_{i}^{-1}\beta_{i}\eta_{i}p_{0}\left(t\right)+\rho_{i}h_{i}p_{0}\left(t\right)\right]dt \end{array}$$

*where*$x_{i}^{*}\left(0\right)=x_{i}^{}\left(0\right)$. $\lambda_{i}\left(t\right)$*is a costate function with*$\lambda_{i}\left(T\right)=0$*and can be given by the following differential equation*:

(9) $$d\lambda_{i}\left(t\right)=\left[-\mu_{i}\left(x_{i}^{*}\left(t\right)-H_{i}z\left(t\right)\right)-\alpha_{i}\lambda_{i}\left(t\right)\right]dt$$

*and*:

$$z\left(t\right)=x^{N}\left(t\right)=\left(1/N\right)\sum_{i=1}^{N}x_{i}\left(t\right)$$

**Theorem** **1.**  *The optimal control problem has a unique solution*.

**Proof.**  The corresponding optimal solution for the SN {i,1≤i≤N} in Equation (7) is given by the Hamilton Jocabi Bellman (HJB) equation, based on the following equation:

(10) $$L_{i}\left(p_{i},x_{i}\right)=\mu_{i}{\left(x_{i}\left(t\right)-H_{i}x^{N}\left(t\right)\right)}^{2}+\nu_{i}{\left(p_{i}\left(t\right)\right)}^{2}+\eta_{i}p_{0}\left(t\right)p_{i}\left(t\right)+\lambda_{i}\left(t\right)\left[\alpha_{i}x_{i}\left(t\right)+\beta_{i}p_{i}\left(t\right)+\rho_{i}h_{i}p_{0}\left(t\right)\right]$$

Then the optimal control problem has a unique solution, which is given by:

(11) $$p_{i}^{*}\left(t\right)=\partial L_{i}\left(p_{i},x_{i}\right)/\partial p_{i}^{}\left(t\right)$$

In Equations (7)–(9), we can find that under the mean field game analysis framework, the corresponding optimal solution for each sensor node can be affected by the mass behaviors of all the sensors. The corresponding optimal solutions can be considered as the mean field game Nash equilibrium control strategies. □

**Proposition**  **2.**  *For each sensor node, the state feedback control equilibrium is given by*:

(12) $$p_{i}^{*}\left(t\right)=\nu_{i}^{-1}\beta_{i}V\left(t\right)x_{i}^{*}\left(t\right)-\nu_{i}^{-1}\beta_{i}\phi_{i}\left(t\right)-\nu_{i}^{-1}\eta_{i}p_{0}\left(t\right)$$

*where*V(t)*is the value function which will be given later. We call Equation (7) or (12) is the optimal localized power strategy of the sensor node*i*for information transmission, because the optimal power strategy is a function of the local information and the strategy of the HAP. In (12), the optimal power strategy*$p_{i}^{*}\left(t\right)$*is a function of the energy state*$x_{i}^{*}\left(t\right)$*and the value function*$V_{i}\left(t\right)$*, where the value function*$V_{i}\left(t\right)$*should satisfy the following relation*:

(13) $$\lambda_{i}\left(t\right)=-V_{i}\left(t\right)x_{i}^{*}\left(t\right)+\phi_{i}\left(t\right)$$

*and*:

(14) $$-\frac{dV_{i}\left(t\right)}{dt}=\nu_{i}^{-1}\beta_{i}^{2}V_{i}{}^{2}\left(t\right)+2\alpha_{i}V\left(t\right)-\mu_{i}$$

*where*$\phi_{i}\left(T\right)=0$*,*$V_{i}\left(T\right)=0$. *Based on Proposition 2, we can obtain the state feedback equilibrium of the optimal control strategy in Equation (7). Meanwhile, the corresponding optimal state trajectory, the corresponding energy variations in Equations (8) and (9) can be re-written as follows*:

(15) $$dx_{i}^{*}\left(t\right)==\left[\left(\alpha_{i}+\beta_{i}^{2}\nu_{i}^{-1}V_{i}\left(t\right)\right)x_{i}^{*}\left(t\right)-\beta_{i}^{2}\nu_{i}^{-1}\phi_{i}\left(t\right)-\beta_{i}\nu_{i}^{-1}\eta_{i}p_{0}\left(t\right)+\rho_{i}h_{i}p_{0}\left(t\right)\right]dt$$

(16) $$d\phi_{i}\left(t\right)==\left[-\left(\alpha_{i}+\beta_{i}^{2}\nu_{i}^{-1}V_{i}\left(t\right)\right)\phi_{i}\left(t\right)+\mu_{i}H_{i}z\left(t\right)-V\left(t\right)\beta_{i}\nu_{i}^{-1}\eta_{i}p_{0}\left(t\right)-\rho_{i}h_{i}p_{0}\left(t\right)\right]dt$$

Next, in order to get the mean field estimation, we should apply the strong law of large numbers (SLLN) to the control strategies given in the above. For each sensor node, the optimal power strategy can be given by Equation (7), and the associated energy state is given by Equation (8). Let $\lambda^{N}\left(t\right)=\left(1/N\right)\sum_{i=1}^{N}\lambda_{i}\left(t\right)$, then $z\left(t\right)=\lim_{N\to \infty }x^{N}\left(t\right)$, and $\lambda \left(t\right)=\lim_{N\to \infty }\lambda^{N}\left(t\right)$ can be given by:

(17) $$dz\left(t\right)=\left[\alpha_{i}z\left(t\right)+\beta_{i}\left(-\beta_{i}\nu_{i}^{-1}\lambda \left(t\right)-\nu_{i}^{-1}\eta_{i}p_{0}\left(t\right)\right)+\rho_{i}h_{i}p_{0}\left(t\right)\right]dt$$

(18) $$d\lambda \left(t\right)=\left[-\mu_{i}\left(z\left(t\right)-H_{i}z\left(t\right)\right)-\alpha_{i}\lambda \left(t\right)\right]dt$$

With the functions given in Equations (15)–(18) can also be written as:

(19) $$dz\left(t\right)==\left[\left(\alpha_{i}+\nu_{i}^{-1}\beta_{i}^{2}V\left(t\right)\right)z\left(t\right)-\beta_{i}^{2}\nu_{i}^{-1}\phi \left(t\right)-\beta_{i}\nu_{i}^{-1}\eta_{i}p_{0}\left(t\right)+\rho_{i}h_{i}p_{0}\left(t\right)\right]dt$$

(20) $$d\phi \left(t\right)==\left[-\left(\alpha_{i}+\beta_{i}^{2}\nu_{i}^{-1}V\left(t\right)\right)\phi \left(t\right)+\mu_{i}H_{i}z\left(t\right)-V\left(t\right)\beta_{i}\nu_{i}^{-1}\eta_{i}p_{0}\left(t\right)-\rho_{i}h_{i}p_{0}\left(t\right)\right]dt$$

where $\phi \left(t\right)=\lim_{N\to \infty }\left(1/N\right)\sum_{i=1}^{N}\phi_{i}\left(t\right)$ and ϕ(T)=0. When the number of the sensor nodes N is arbitrary large, we can find the mean field estimation based on Equation (19) and (20). Additionally, we can find that the mean field estimation is dependent on the HAP’s power control strategy.

**Proposition**  **3.**  *For any power strategy of the HAP, we have*:

(21) $$Ε\int_{0}^{T}{\left\|x^{N}\left(t\right)-H_{i}z\left(t\right)\right\|}^{2}dt=O\left(\frac{1}{N}\right)$$

*Recall the optimal power strategy for the sensor node*i*,*1≤i≤N, *in Proposition 1*:

(22) $$p_{i}^{*}\left(t\right)=-\nu_{i}^{-1}\beta_{i}\lambda_{i}\left(t\right)-\nu_{i}^{-1}\eta_{i}p_{0}\left(t\right)$$

*where*$\left(x_{i}^{*},\lambda_{i}\right)$*is determined in Equations (8) and (9). The above optimal control strategy given in Equation (22) is an open loop solutions controlled by the power control strategy of the HAP*.

**Theorem** **2.**  *For any power strategy of the HAP for energy transfer, the information transmission strategy for each sensor node*{i,1≤i≤N}*, constitutes an*ε*-Nash equilibrium, that is, for any*i*,*1≤i≤N, *we have*:

(23) $$J_{i}^{N}\left({\bar{p}}_{i},{\bar{p}}_{-i},p_{0}\right)\leq \inf_{p_{i}\in U_{i}\left(p_{0}\right)}J_{i}^{N}\left(p_{i},{\bar{p}}_{-i},p_{0}\right)+\varepsilon$$

*where*$\varepsilon =O\left(1/\sqrt{N}\right)$.

### 4.2. Mean Field Control of HAP

In this section, we will analyze the mean field control problem for the HAP, and try to obtain the optimal control strategy. The open-loop solution will be given and the mean field control solution can be obtained.

**Definition** **2.**  *For the HAP, the control strategy*$p_{0}^{*}\left(t\right)$*are optimal if the following inequality holds for all feasible controls*$p_{0}^{}\left(t\right)\neq p_{0}^{*}\left(t\right)$:

(24) $$J_{0}^{N}\left(p_{0}^{*},p^{N}\right)\leq J_{0}^{N}\left(p_{0},p^{N}\right)$$

**Proposition** **4.**  *The HAP’s optimal control problem is to minimize the following equation*:

(25) $$J_{0}^{N}\left(p_{0},p^{N}\right)=Ε\left(\int_{0}^{T}\left\{\mu_{0}{\left(x_{0}\left(t\right)-H_{0}z\left(t\right)\right)}^{2}+\nu_{0}{\left(p_{0}\left(t\right)\right)}^{2}\right\}dt\right)$$

*subject to*:

(26) $$dx_{0}\left(t\right)=\left[\alpha_{0}x_{0}\left(t\right)+\beta_{0}p_{0}\left(t\right)\right]dt$$

(27) $$dz\left(t\right)=\left[\alpha_{i}z\left(t\right)+\beta_{i}\left(-\nu_{i}^{-1}\beta_{i}\lambda \left(t\right)-\nu_{i}^{-1}\eta_{i}p_{0}\left(t\right)\right)+\rho_{i}h_{i}p_{0}\left(t\right)\right]dt$$

(28) $$d\lambda \left(t\right)=\left[-\mu_{i}\left(z\left(t\right)-H_{i}z\left(t\right)\right)-\alpha_{i}\lambda \left(t\right)\right]dt$$

As the HAP is the leader in the proposed game model, and we should apply the Stackelberg game analysis to the proposed model, there exists two more constraints in the control of the HAP compared to the control of the sensor nodes, given by Equations (27) and (28). Based on the mean field control solutions given in Section 3, the constraints given by Equations (27) and (28) can be replaced by:

(29) $$dz\left(t\right)==\left[\left(\alpha_{i}+\nu_{i}^{-1}\beta_{i}^{2}V\left(t\right)\right)z\left(t\right)-\beta_{i}^{2}\nu_{i}^{-1}\phi \left(t\right)-\beta_{i}\nu_{i}^{-1}\eta_{i}p_{0}\left(t\right)+\rho_{i}h_{i}p_{0}\left(t\right)\right]dt$$

(30) $$d\phi \left(t\right)==\left[-\left(\alpha_{i}+\beta_{i}^{2}\nu_{i}^{-1}V\left(t\right)\right)\phi \left(t\right)+\mu_{i}H_{i}z\left(t\right)-V\left(t\right)\beta_{i}\nu_{i}^{-1}\eta_{i}p_{0}\left(t\right)-\rho_{i}h_{i}p_{0}\left(t\right)\right]dt$$

**Proposition** **5.**  *For the HAP, there exists an optimal control solution given by the pair*$\left(x_{0}^{*},p_{0}^{*}\right)$*if and only if*

(31) $$p_{0}^{*}\left(t\right)=-\nu_{0}^{-1}\beta_{0}\lambda_{0}\left(t\right)+\nu_{0}^{-1}\Lambda_{1}\left(t\right)\sum_{i=1}^{N}\eta_{i}\nu_{i}^{-1}\beta_{i}-\nu_{0}^{-1}\Lambda_{2}\left(t\right)\sum_{i=1}^{N}\rho_{i}h_{i}$$

*where*:

(32) $$dx_{0}\left(t\right)=\left[\alpha_{0}x_{0}\left(t\right)-\nu_{0}^{-1}\beta_{0}^{2}\lambda_{0}\left(t\right)+\nu_{0}^{-1}\beta_{0}\Lambda_{1}\left(t\right)\sum_{i=1}^{N}\eta_{i}\nu_{i}^{-1}\beta_{i}-\nu_{0}^{-1}\beta_{0}\Lambda_{2}\left(t\right)\sum_{i=1}^{N}\rho_{i}h_{i}\right]dt$$

(33) $$d\lambda_{0}=\left[-\mu_{0}\left(x_{0}^{*}\left(t\right)-H_{0}z\left(t\right)\right)-\alpha_{0}\lambda_{0}\left(t\right)\right]dt$$

(34) $$d\Lambda_{1}=\left[\eta_{i}H_{0}\left(x_{0}^{*}\left(t\right)-H_{0}z\left(t\right)\right)-\Lambda_{1}\sum_{i=1}^{N}\alpha_{i}-\Lambda_{2}\sum_{i=1}^{N}\eta_{i}\left(H_{i}-1\right)\right]dt$$

(35) $$d\Lambda_{2}=\left[\Lambda_{1}\sum_{i=1}^{N}\beta_{i}^{2}\nu_{i}^{-1}+\Lambda_{2}\sum_{i=1}^{N}\alpha_{i}\right]dt$$

(36) $$\begin{array}{l} dz\left(t\right)=\left[\begin{array}{l} \alpha_{i}z\left(t\right)-\nu_{i}^{-1}\beta_{i}^{2}\lambda_{i}\left(t\right)-\nu_{i}^{-1}\beta_{i}\eta_{i}\left[\begin{array}{l} -\nu_{0}^{-1}\beta_{0}\lambda_{0}\left(t\right)+\nu_{0}^{-1}\Lambda_{1}\left(t\right)\sum_{i=1}^{N}\eta_{i}\nu_{i}^{-1}\beta_{i}\\ -\nu_{0}^{-1}\Lambda_{2}\left(t\right)\sum_{i=1}^{N}\rho_{i}h_{i} \end{array}\right]\\ +\rho_{i}h_{i}\left[-\nu_{0}^{-1}\beta_{0}\lambda_{0}\left(t\right)+\nu_{0}^{-1}\Lambda_{1}\left(t\right)\sum_{i=1}^{N}\eta_{i}\nu_{i}^{-1}\beta_{i}-\nu_{0}^{-1}\Lambda_{2}\left(t\right)\sum_{i=1}^{N}\rho_{i}h_{i}\right] \end{array}\right]dt \end{array}$$

(37) $$d\lambda \left(t\right)=\left[-\mu_{i}\left(z\left(t\right)-H_{i}z\left(t\right)\right)-\alpha_{i}\lambda \left(t\right)\right]dt$$

### 4.3. Mean Field Control Algorithm

**Figure 4:**
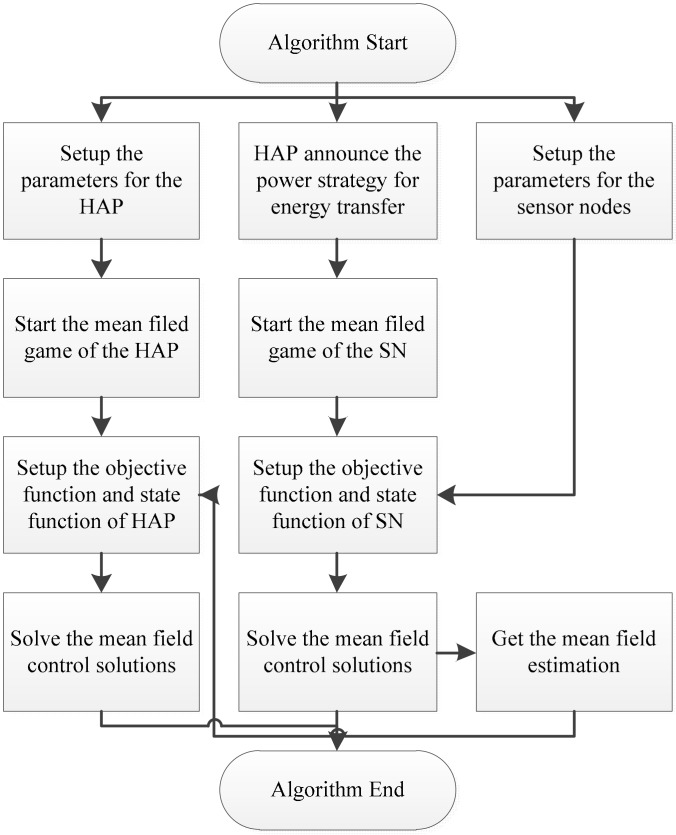
Implementation algorithm.

In this subsection, we will discuss the implementation algorithm for the proposed model. As shown in Figure 4, the whole algorithm cycling can be divided into two parts. One is the “mean field control of sensor nodes” part, which is used to calculate the equilibrium for the sensor nodes. The other is the “mean field control of HAP” part, to make a decision on the power level for energy transfer. As all the objective functions given in the mean field control process are linear quadratic functions, and the solutions should be solved based on the Stackelberg game framework, the complexity of the algorithm will be $O\left(n^{2}\right)$. The progress can be described as follows.

**Algorithm 1** Mean field control algorithm for the HAP and sensor nodes.
Set up the parameter for the HAP and sensor nodes.The HAP announce the power strategy for energy transfer to the sensor nodes.Start the mean field game of the HAP and sensor nodes.Calculate the mean field control solutions for the sensor nodes first. Setup the objective function and state function for the sensor nodes. Calculate the solutions for the sensor nodes based on Equations (12)–(18).Get the mean field estimation of the sensor nodes for the HAP.Calculate the mean field control solutions for the HAP. Setup the objective function and state function for the HAP. Calculate the solutions for the HAP based on Equations (31)–(37).End.


## 5. Performance Evaluation

In this section, we provide simulation results to illustrate the convergence property and effectiveness of the proposed model. Assuming all the sensor nodes are uniform sensor node, that have the same parameter settings. Each sensor wants to control the power level for information transmission to minimize the cost given in Equation (5). The mean field control solutions introduced in Section 3 and Section 4 are simulated. 

**Figure 5:**
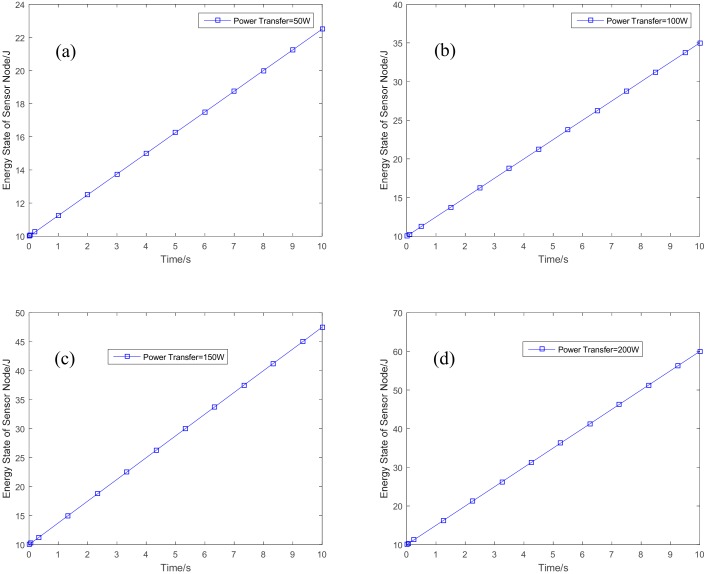
Variations of energy state with different transfer power. (**a**) Power transfer = 50 W; (**b**) power transfer = 100 W; (**c**) power transfer = 150 W; and (**d**) power transfer = 200 W.

**Figure 6:**
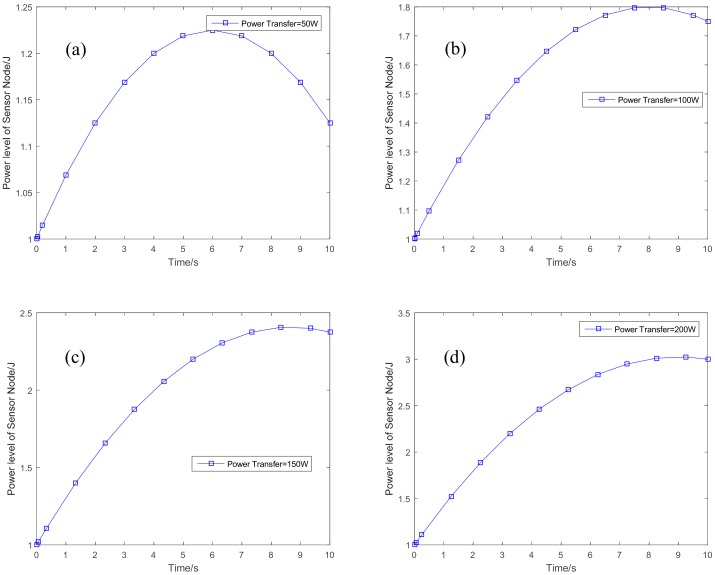
The power level for information transmission. (**a**) Power transfer = 50 W; (**b**) power transfer = 100 W; (**c**) power transfer = 150 W; and (**d**) power transfer = 200 W.

Figure 5 shows the optimal variations of the energy state for the sensor node, with the power level for energy transfer are set to be 50 W, 100 W, 150 W, and 200 W, respectively. In Figure 5a, the power level for energy transfer is set to be 50 W, the energy state of the sensor node can be increased from the initial energy state to a higher energy state with energy transfer. When we increase the power level of the HAP for energy transfer in Figure 5b–d, the final energy state will be increased. The higher of the transfer energy, the higher of the achieved energy state. The sensor node can have much more energy stored in its battery with higher energy from the HAP. Related to the energy state, the power level for information transmission for each sensor node is given in Figure 6. With the increasing of the transferred energy, there will be more power for the sensor node to transmit information. When the power level of HAP for energy transfer is 50 W, shown in Figure 6a, the sensor node will increase the power level for information transmission at the first 6 s. It will decrease the power level to have more energy available at the next 4 s. The power level for the information transmission can achieve convergence when the power level for energy transfer is large than 50 W.

**Figure 7:**
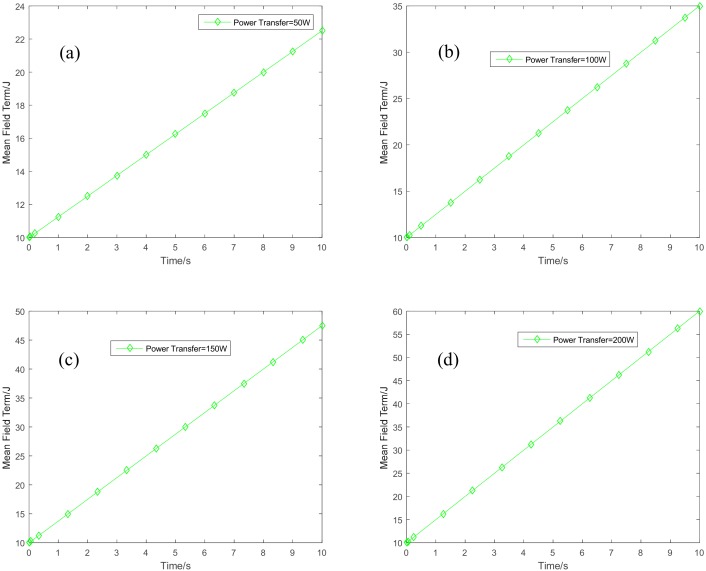
Variations of the mean field term. (**a**) Power transfer = 50 W; (**b**) power transfer = 100 W; (**c**) power transfer = 150 W; and (**d**) power transfer = 200 W.

**Figure 8:**
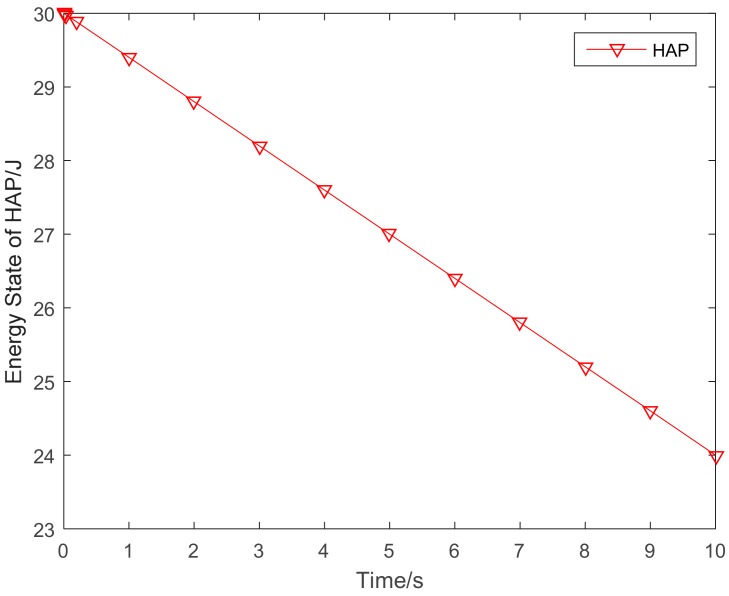
Variations of the HAP’s energy.

The variation of the mean filed term, the mass behavior of the sensor nodes is given in Figure 7. Figure 8 shows the variation of the energy state of the HAP. As the HAP is the energy source for the sensor nodes, its energy will decrease with the time duration.

## 6. Conclusions

In this paper, we have proposed a Stackelberg mean field game-based model to solve the power control problems in the wireless powered IoT system, to minimize the cost of the information transmission for the sensor nodes, and to minimize the cost of the HAP. In the proposed game model, the relations between the HAP and sensors is analyzed based on the Stackelberg game, and the objective functions are constructed using the mean field game. We consider the energy variations of sensors and HAP as the system state to construct the mean field game model. Then mean filed control for both the sensor nodes and the HAP are analyzed, and ε-Nash equilibriums are obtained. Based on the simulations results, it can be seen that our proposed model can achieve optimal power control for both the sensor nodes and the HAP. In future work, we will attempt to extend our proposed mean field Stackelberg game-based algorithm, in order to employ it in other kinds of networks, such as smart grid networks [25], M2M networks [26], 5G networks [27,28], and so on [29].
